# Monogenetic Forms of Parkinson’s Disease – Bridging the Gap Between Genetics and Biomarkers

**DOI:** 10.3389/fnagi.2022.822949

**Published:** 2022-03-03

**Authors:** Lars Tönges, Eun Hae Kwon, Stephan Klebe

**Affiliations:** ^1^Department of Neurology, St. Josef-Hospital, Ruhr-University Bochum, Bochum, Germany; ^2^Center for Protein Diagnostics (ProDi), Ruhr University Bochum, Bochum, Germany; ^3^Department of Neurology, University Hospital Essen, Essen, Germany

**Keywords:** genetic, biomarkers, Parkinson’s disease, alpha-synuclein, LRRK2

## Abstract

The therapy of neurodegenerative diseases such as Parkinson’s disease (PD) is still limited to the treatment of symptoms and primarily aimed at compensating for dopaminergic hypofunction. Numerous disease-modifying therapies currently in the pipeline attempt to modify the underlying pathomechanisms. In recent decades, the results of molecular genetics and biomarker research have raised hopes of earlier diagnosis and new neuroprotective therapeutic approaches. As the disease-causing processes in monogenetic forms of PD are better understood than in sporadic PD, these disease subsets are likely to benefit first from disease-modifying therapies. Recent studies have suggested that disease-relevant changes found in genetically linked forms of PD (i.e., PARK-LRRK2, PARK-GBA) can also be reproduced in patients in whom no genetic cause can be found, i.e., those with sporadic PD. It can, therefore, be assumed that as soon as the first causal therapy for genetic forms of PD is approved, more patients with PD will undergo genetic testing and counseling. Regarding future neuroprotective trials in neurodegenerative diseases and objective parameters such as biomarkers with high sensitivity and specificity for the diagnosis and course of the disease are needed. These biomarkers will also serve to monitor treatment success in clinical trials. Promising examples in PD, such as alpha-synuclein species, lysosomal enzymes, markers of amyloid and tau pathology, and neurofilament light chain, are under investigation in blood and CSF. This paper provides an overview of the opportunities and current limitations of monogenetic diagnostic and biomarker research in PD and aims to build a bridge between current knowledge and association with PD genetics and biomarkers.

## Introduction

Parkinson’s disease (PD) [sporadic (sPD) and (mono)genetic forms of PD (gPD)] represents a heterogeneous group of disorders with the pathophysiological shared end of a dopaminergic deficit. Therapy of PD is still limited to the treatment of symptoms and is primarily aimed at compensating for dopaminergic hypofunction. Accurate diagnosis of PD according to the underlying pathophysiology is important because of current and future treatment options. In recent years, many efforts have been made to identify specific and sensitive biomarkers (Parnetti et al., 2019; Lawton et al., 2020). Because of its proximity to neuronal structures, cerebrospinal fluid (CSF) is, of course, the most promising liquid biomarker for neurodegenerative diseases. Nevertheless, blood, saliva, skin biopsies, and urine also appear to be good candidates as they are easily accessible and less invasive. Biomarkers from body fluids, skin biopsies, or imaging studies have the advantage of identifying the risk for future PD, diagnosing PD early, and monitoring disease progression, and can serve to monitor possible treatment success in clinical trials. Hence, biomarkers should also be able to distinguish between different entities of neurodegenerative diseases [e.g., sPD vs. gPD vs. atypical Parkinson’s syndromes (aPD)] to decrease the number of false-positive diagnosed patients. An unmet need for future neuroprotective trials in neurodegenerative diseases is an objective parameter such as biomarkers with high specificity and sensibility for the diagnosis and course of the disease. Promising examples in PD, such as alpha-synuclein species, lysosomal enzymes, markers of amyloid and tau pathology, and neurofilament light chain, are under investigation in the blood and CSF (Parnetti et al., 2019). Unfortunately, it has not been possible to date to implement a robust biomarker in the early diagnosis or follow-up of PD. One reason is that biomarker profiles vary from patient to patient due to the great heterogeneity of the disease.

In contrast to sPD, in which the etiology remains elusive for most cases, gPD offer the possibility of better understanding the cause of the disease. Many autosomal dominant and recessive genes have been found since the first description of *SNCA* as a genetic cause of PD in 1997 (Polymeropoulos et al., 1997). The clinical symptoms between sPD and a monogenetic form are similar in most cases. However, since the course of PD can differ greatly from individual to individual, the question arises whether the etiological heterogeneity might have different causes. Examples include the tremor-dominant or akinetic rigid subtypes of sPD or the early cognitive involvement in some patients with PD. The results of molecular genetics and biomarker research have raised hopes of resolving these unanswered questions in PD and finding new neuroprotective therapeutic approaches. As the disease-causing processes in gPD are better understood than in sPD, patients with these subsets of the disease are likely to benefit first from disease-modifying therapies. A synergistic effect between research on monogenetic forms of the disease and sPD is also likely. Recent studies have suggested that disease-relevant changes found in genetically linked forms of PD (i.e., PARK-LRRK2, PARK-GBA) can also be reproduced in patients in whom no genetic cause can be found (Di Maio et al., 2018). Even if these patients with gPD currently represent only a small group (approximately 10%) (Cook et al., 2021), it is expected that availability of a causal therapy for genetic forms of PD will substantially increase demand for genetic testing and counseling. Therefore, we aim to provide an overview of the most common genetically linked forms of PD and the associated biomarker studies. Table 1 summarizes the genetic causes of PD with regard to the corresponding biomarker.

**TABLE 1:** An overview of the genetic causes of Parkinson’s disease (PD), the corresponding biomarker, and the diagnostic and/or prognostic value.

Genetics	Source	Biomarker	Findings (diagnostic/prognostic*)	Study
*SNCA*	Blood	Oligomeric aSyn	↑sPD vs. HC (sensitivity 75%; specificity 100%)	Williams et al., 2016
	CSF	Total aSyn	↓sPD vs. HC	Mollenhauer et al., 2008
			↑PARK-LRRK2/HC vs. sPD	Vilas et al., 2016
		Oligomeric aSyn	↑sPD vs. HC (sensitivity 71%; specificity 64%)	Eusebi et al., 2017
			↑sPD vs. HC (RT-QuIC) (sensitivity 95%; specificity 100%)	Fairfoul et al., 2016; Sano et al., 2018; Shahnawaz et al., 2020
		Phosphorylated aSyn	↑sPD vs. HC vs. PSP	Eusebi et al., 2017
*GBA*	CSF	GCase activity	↓sPD vs. HC	Parnetti et al., 2014a; Lerche et al., 2021
			↓PARK-GBA vs. HC	Xicoy et al., 2019
			↓PARK-LRRK2 vs. HC	
			↓PARK-PRKN vs. HC	
	Blood		Positive correlation with disease duration*	Kim et al., 2016
*LRRK2*	Blood	Total LRRK2	↑sPD vs. HC	Atashrazm et al., 2019
			sPD = PARK-LRRK2/HC	Padmanabhan et al., 2020
		pS935-LRRK2	↓PARK-LRRK2 vs. sPD/HC	Padmanabhan et al., 2020
			↑sPD vs. HC	Melachroinou et al., 2020
		pRab10	PARK-LRRK2 = sPD/HC	Atashrazm et al., 2019
	CSF	Total LRRK2	↑PARK-LRRK2 vs. sPD/HC	Mabrouk et al., 2020
		pS1292-LRRK2	PARK-LRRK2 = sPD/HC	Wang et al., 2017
		Aβ1-42, total-, phospho-Tau	PARK-LRRK2 = sPD/HC	Vilas et al., 2016
	Urine	pS1292/total LRRK2	↑PARK-LRRK2 vs. sPD/HC	Fraser et al., 2016
	Neuro-imaging	PET multitracer	↓LRRK2 vs. controls	Nandhagopal et al., 2008
		I-123 FP-CIT SPECT	LRRK2 PD conversion rate = 16%*	Sánchez-Rodríguez et al., 2021
*PRKN/PINK1*	Blood	pS65-Ub	↓PARK-PINK1 vs. HC	Watzlawik et al., 2020
		Ccf-mtDNA	↑PARK-PRKN/PINK1 vs. sPD/HC	Borsche et al., 2020
		IL6	↑sPD vs. HC	Qin et al., 2016
			Positive correlation with disease severity*	Green et al., 2019
			↑PARK-PRKN/PINK1 vs. sPD vs. HC	Borsche et al., 2020
	CSF	Ccf-mtDNA	↓sPD vs. HC	Pyle et al., 2015
	Neuro-imaging	PET 18F-BCPP-EF tracer	sPD = HC	Wilson et al., 2020
		31P-MR-spectroscopy	↑PARK-PINK1 vs. HC	Hilker et al., 2012
*DJ-1*	CSF	DJ1	Conflicting results in sPD	
	Saliva		Conflicting results in sPD	
	Urine	Oxidized DJ1	↑sPD vs. controls	Jang et al., 2018

aSyn, alpha-synuclein; CSF, cerebrospinal fluid; GCase, activity of the lysosomal hydrolase ß-glucocerebrosidase; HC, healthy controls; IL6, interleukin 6; PET, Positron emission tomography; RT-QuIC, real-time quaking-induced conversion; sPD, sporadic Parkinson’s disease; SPECT, single photon emission computed tomography; ↑, increased levels; ↓, decreased levels; *, prognostic.

## Alpha-Synuclein

Alpha-synuclein (aSyn) is a rather small protein (14 kDa) that is widely expressed in the brain (Braak et al., 2003). It seems to play a role in the stability of neuronal membranes, influencing presynaptic signaling and membrane trafficking through vesicular transport (Henderson et al., 2019). Several papers show that aSyn can be released from neurons and taken up by surrounding neurons or other cell types (Henderson et al., 2019). Therefore, it is not surprising that aSyn, despite constituting an intracellular protein, can be found in CSF, blood, and plasma (Atik et al., 2016; Maass et al., 2019). aSyn aggregates represent the main component of Lewy bodies (LB) (Spillantini et al., 1997). Therefore, PD, together with dementia with Lewy bodies (DLB) and multiple system atrophy (MSA), is referred to as a-synucleinopathy. LB are found in various regions of the CNS, depending on the disease stage (for example, substantia nigra, the dorsal motor nucleus of the vagus, nucleus basalis of Meynert, the locus coeruleus, and diffusely in late stages of the disease) (Forno, 1996). However, LB are also found in the peripheral nervous system in early or prodromal stages of the disease (e.g., sympathetic ganglia, sympathetic cardiac nerves, cholinergic nerve endings of salivary glands, autonomic nerve fibers of the skin).

First described with point mutations, the gene for aSyn (*SNCA*) was the first gene to be associated with the development of PD with an autosomal-dominant transmission (Polymeropoulos et al., 1997). Over time, duplications and triplications of *SNCA* have also been shown to play a causal role in hereditary forms of PD. Regarding symptoms, *SNCA* point mutations are associated with an earlier age at the onset (Ibáñez et al., 2009). *SNCA* gene duplications are associated with a mean age of the onset and clinical phenotype that overlaps with sPD (Ibáñez et al., 2009). *SNCA* triplications are associated with the early age of the onset, more rapid progression, and memory impairment (Ibáñez et al., 2009). Nonetheless, mutations in *SNCA* as a disease cause in PD remain rare.

Total aSyn and isoforms of aSyn appear to play a major role in PD pathogenicity and, accordingly, are also available as a candidate biomarker. The concentration of total aSyn in CSF may discriminate between patients with PD and healthy controls (Wang et al., 2012), and has been found at a lower concentration in patients with PD (Mollenhauer et al., 2008). This was confirmed in early-diagnosed, treatment-naïve patients (Stewart et al., 2014).

However, no uniform picture emerges regarding total aSyn. In patients with *LRRK2* mutations, total aSyn CSF levels were increased in *LRRK2*-associated PD, non-manifesting carriers, and healthy controls compared to sPD (Vilas et al., 2016). In another study, lower but not statistically significant total aSyn CSF levels were recognized in *LRRK2*-associated PD, non-manifesting carriers, and sPD compared to healthy controls (Aasly et al., 2014). Follow-up data in patients with PD from the DATATOP study revealed an increase of total aSyn after 2 years (Majbour et al., 2016). An increase in total aSyn has also been reported over 2 years in another study with 63 patients with PD and 21 controls (Hall et al., 2016). In the latter study, higher total aSyn values were associated with longer disease duration (Hall et al., 2016). The authors hypothesized that total aSyn levels were bimodal, with a decrease in early disease stages followed by an increase with advanced neurodegeneration.

Unfortunately, unlike MSA or progressive supranuclear palsy (PSP), it is not possible to distinguish between sPD and aPD based on the aSyn concentration. Therefore, total aSyn is unable to distinguish between alpha-synucleinopathies (sPD, MSA) or between alpha-synucleinopathies and aPD such as PSP or corticobasal degeneration. Because of the cognitive impairment in *SNCA* triplication carriers, an association between total aSyn and patients with PD with dementia (PDD) could be considered. However, similar levels of total aSyn have been reported for patients with PD and PDD (Farotti et al., 2017).

The reason for the large heterogeneity of the results with respect to total aSyn is likely due to clinical heterogeneity, the number of included patients, and methodological differences that could compromise diagnostic accuracy. Another important confounding variable is hemolysis during the specimen collection, where contamination of blood or CSF leads to increased total aSyn levels (Barkovits et al., 2020).

Before aSyn aggregates into mature amyloid fibrils in LB, aSyn undergoes oligomerization. There is strong evidence that oligomeric aSyn is a key player in PD pathophysiology (Conway et al., 2000). It has been found at higher concentration in patients with PD compared to controls, but with poor diagnostic sensitivity and specificity (Parnetti et al., 2014b; Eusebi et al., 2017). However, there are conflicting results with respect to oligomeric aSyn reported over recent years. One example is the results in asymptomatic and symptomatic PARK-LRRK2 mutation carriers. Asymptomatic PARK-LRRK2 mutation carriers showed elevated oligomeric aSyn levels compared to healthy controls (Aasly et al., 2014); that oligomeric aSyn reflects a presymptomatic precursor of the disease cannot be concluded with certainty because symptomatic PARK-LRRK2 mutation carriers in the same study showed no difference to healthy controls (Aasly et al., 2014). Again, there is no evidence that oligomeric aSyn in the CSF might distinguish between sPD, gPD, and/or aPD. Among the isoforms of aSyn, phosphorylated 129 aSyn has been reported to enhance synuclein toxicity both *in vivo* and *in vitro* due to increasing formation of aSyn aggregates (Fujiwara et al., 2002; Smith et al., 2005), whereas a protective effect of phosphorylated 129 aSyn has also been described (Gorbatyuk et al., 2008; Da Azeredo Silveira et al., 2009). Another biomarker could be the 129 aSyn/total aSyn ratio, which seems to be elevated in PD (Wang et al., 2012). Regarding aSyn aggregates, *in vitro* conversion methods such as real-time quaking-induced conversion (RT-QuIC) have been developed as promising new methods for measuring aggregated aSyn in the CSF of patients with PD. RT-QuIC was originally developed for the detection of the abnormal form of prion protein, e.g., in Creutzfeldt-Jakob disease. In alpha-synucleinopathies, RT-QuIC has been reported with a high sensitivity and specificity of 95-100% for alpha-synucleinopathies (Fairfoul et al., 2016; Sano et al., 2018; Shahnawaz et al., 2020) and a positive predictive value of > 90% (van Rumund et al., 2019), but only 75% sensitivity in patients with an unclear diagnosis of parkinsonism (van Rumund et al., 2019). In addition, RT-QuIC does not appear to be suitable for monitoring the disease course (Nakagaki et al., 2021). The method has also been used to distinguish between alpha-synucleinopathies and other neurodegenerative diseases (Groveman et al., 2018). Due to an improved aSyn RT-QuIC test, a diagnostic sensitivity of 93% and 100% specificity has been achieved when comparing nine patients with synucleinopathy (12 PD; 17 DLB) and 31 non-synucleinopathy controls (including 16 patients with Alzheimer’s diseases). A recent study has presumed that RT-QuIC of aSyn in the CSF could be a promising candidate for prodromal stages for PD and DLB. In patients with isolated REM sleep behavior disorder (RBD), a known risk factor in the development of PD and DLB, a sensitivity and specificity of 90% have been shown (Iranzo et al., 2021). In addition, these patients had a subsequent higher risk of developing PD or DLB. Nearly two-thirds of the patients with RBD were diagnosed with PD or DLB, a mean 3.4 years after the aSyn measurement, of whom 97% were aSyn positive at the baseline (Iranzo et al., 2021). This finding of aSyn in the CSF by RT-QuIC and its value in presymptomatic patients is important, but must be confirmed by further studies.

The different forms of aSyn (total aSyn, oligomeric aSyn, phosphorylated aSyn) also reveal different results in blood serum and plasma. For total aSyn, conflicting results of increased, decreased, or unchanged total aSyn levels have been reported (Parnetti et al., 2019). Oligomeric aSyn in blood serum or red blood cells seems valuable candidates, with a sensitivity of 75% and specificity of up to 100% (Williams et al., 2016). However, these results must be confirmed in larger studies.

## ß-Glucocerebrosidase

Lysosomes are one of the key players for protein degradation in human cells, including a-Syn (Webb et al., 2003). It is obvious that lysosome malfunction leads to an accumulation of dysfunctional proteins and cell organelles. This pathway is shared by several monogenetic forms of PD, including *SNCA*, *ATP13A2*, *VPS35*, *DNAJC6*, *SYNJ1*, *LRRK2*, *RAB39B9*) (Abeliovich and Gitler, 2016). An association between aSyn and lysosomes has long been hypothesized. It is assumed that a disturbed autophagy-lysosomal pathway is directly related to aSyn aggregates (Sardi et al., 2011; Bae et al., 2015; Suzuki et al., 2015).

Mutations in the corresponding gene for ß-glucocerebrosidase (*GBA*) are considered the most common genetic risk factor in PD, meaning that not every carrier of a *GBA* mutation will develop PD. Historically, the observation of an increased risk of PD in type I Gaucher disease (GD) and its families was first surmised from clinical findings (Neudorfer et al., 1996). GD type I represents an autosomal-recessive systemic disorder that can present with various degrees of systemic and neurological manifestations, usually manifesting in childhood. Since the first description, more than 300 different genetic alterations, such as point mutations, insertion, deletion, missense mutations, and splice junctions, have been described in the literature (Beutler et al., 2005; Hruska et al., 2008; Smith et al., 2017). Among GBA mutation carriers, an increasing penetrance of PD (PARK-GBA) with age has been reported (Anheim et al., 2012). Penetrance ranges from 7.6% at age 50 years to 30% at age 80 (Anheim et al., 2012). If patients have two homozygous mutations and thus affected with GD, the risk of PD is higher and the age at the onset (AAO) is earlier (Thaler et al., 2017). Depending on the origin of the patients, the frequency of *GBA* mutations varies. For example, *GBA* mutations can be found in 10–31% of patients with Ashkenazi background but only in 2.3% of Norwegian patients (Neumann et al., 2009). For the European non-Ashkenazi patients, the range is between 3 and 12% (Neumann et al., 2009).

Clinical differentiation between sPD and gPD caused by a heterozygous *GBA* mutation (PARK-GBA) is not possible. However, the AAO of PARK-GBA is 3–6 years earlier than in sPD (Riboldi and Di Fonzo, 2019). The progression of PARK-GBA is more often characterized by prominent cognitive impairment, hallucination, and RBD. Due to the phenotypic description, it is not surprising that *GBA* mutations have also been found in cases of DLB (Nalls et al., 2013). The frequency of patients with DLB with *GBA* mutations was up to 13% in pathologically proven patients with DLB (Gámez-Valero et al., 2016).

A few studies have focused on the activity of the lysosomal hydrolase ß-glucocerebrosidase (GCase) in alpha-synucleinopathies, with a significant decrease of GCase activity in the CSF of patients with PD compared to controls (Parnetti et al., 2014a; Lerche et al., 2021). As a biomarker decreased, GCase activity was consistently found in PARK-GBA (see below) but also in other gPD (PARK-LRRK2, PARK-SNCA, PARK-RKN) (Xicoy et al., 2019). The overall utility of GBA as biomarkers in peripheral biosamples of sPD is highly controversial because, as with the results in CSF, dried blood spots are conflicting (Balducci et al., 2007; Parnetti et al., 2017; Pchelina et al., 2017). In a very recently published work, the authors have hypothesized that low GCase activity was not responsible for the phenotype because low GCase activity has been found in patients with PARK-GBA and non-manifesting GBA carriers (Omer et al., 2022). However, patients with PARK-GBA were nearly 10 years older than non-manifesting GBA carriers (64.9 vs. 53.4 years, respectively). An additional role could be the respective GBA mutation, as there are indications that this causes different changes in GCase activity (Lerche et al., 2021). GCase activity could also be promising as a progression parameter in leukocytes, as a positive correlation between GCase levels in leukocytes and disease duration has been reported in sPD (Kim et al., 2016). Unfortunately, the treatments currently available for patients with GD do not reach the CNS. Hence, these treatment options do not play a role in PARK-GBA. However, there are several approaches to therapy development. One example is the capacity to increase GCase levels, currently being tested using the chaperone ambroxol in patients with PARK-GBA in an ongoing study (NCT02914366) (Mullin et al., 2020). The rationale behind this is that mutant *GBA* is unable to fold the endoplasmic reticulum correctly and thus promotes protein aggregation. Chaperone proteins able to facilitate the refolding of their substrates were tested. Another approach is gene replacement therapy *via* adeno-associated virus 9 to deliver a functional copy of the *GBA* gene to the CNS (Du et al., 2019; Abeliovich et al., 2021). In several animal models of PD or *GBA*-associated PD, direct application to the CNS was successful (Rocha et al., 2015; Jackson et al., 2019). Accordingly, a phase 1/2a study using these techniques has been recently started (NCT04127578).

## Leucine-Rich Repeat Kinase

Mutations in the leucine-rich repeat kinase (*LRRK2*) gene are the most common genetic cause of autosomal-dominant late-onset PD (Paisán-Ruíz et al., 2004; Zimprich et al., 2004). *LRRK2* variants are also considered significant risk factors in sPD cases (Kluss et al., 2019). G2019S is the most frequent variant, accounting for 4% of gPD and 1% of sPD, with variable distribution among different ethnic populations and incomplete, age-related penetrance (26–84%) (Healy et al., 2008; Lee et al., 2017). *LRRK2* encodes a multifunctional protein with a catalytic core of kinase and GTPase activity. Physiologically, LRRK2 is involved in various cellular processes, encompassing cytoskeletal maintenance, vesicular trafficking, mitochondrial function, autophagy, lysosomal degradation, and the inflammatory response (Jeong and Lee, 2020; Mancini et al., 2020). Most *LRRK2* mutations cause a toxic gain of function with increased kinase activity. In viral and transgenic rodent models, overexpression of *LRRK2* G2019S induced striatal neurodegeneration in a kinase-dependent manner (Lee et al., 2010; Tsika et al., 2015). There is also evidence that LRRK2 interacts with aSyn, mediating its aggregation and propagation (O’Hara et al., 2020).

The phenotype of PARK-LRRK2 resembles that of sPD in terms of cardinal motor features and good response to levodopa (Healy et al., 2008; Marras et al., 2011). PD symptoms manifest at a slightly younger age (57 years). Several studies have observed a more benign disease course in G2019S mutation carriers with a slower decline in motor scores, albeit a similar risk for motor complications (Healy et al., 2008; Marras et al., 2016; Ben Romdhan et al., 2018; Saunders-Pullman et al., 2018) that could not be confirmed by other studies that report no difference (Nabli et al., 2015) or even worse motor symptoms and a higher rate of dyskinesia (Nishioka et al., 2010; Shu et al., 2018). Various studies have indicated that PARK-LRRK2 is associated with less non-motor involvement. Hyposmia and RBD are less prevalent compared to sPD (Saunders-Pullman et al., 2015; Marras et al., 2016), and cognitive function seems to be more mildly impaired, even after many years of disease duration (Aasly et al., 2005; Alcalay et al., 2015).

Given the therapeutic advances of small molecule kinase inhibitors and antisense oligonucleotides to diminish LRRK2 activity, reliable and feasible biomarkers that reflect LRRK2-related pathways are mandatory. LRRK2 is highly expressed in peripheral tissues, such as lung, kidney, and blood cells (Fuji et al., 2015), and the latter serves as an easily accessible source [i.e., peripheral blood mononuclear cells (PBMCs)]. Indeed, by using flow cytometry, significantly elevated LRRK2 levels have been detected in B cells, T cells, CD16 + monocytes (Cook et al., 2017), and neutrophils of subjects with sPD compared to controls (Atashrazm et al., 2019), supporting the link to immune regulation. However, no difference in total LRRK2 levels could be detected in PBMCs among various sample groups, irrespective of PD or *LRRK2* mutation status (Melachroinou et al., 2020; Padmanabhan et al., 2020). Alterations of total LRRK2 levels in PBMCs could be obscured by cellular heterogeneity. Considering cell-specific expression patterns of LRRK2, it seems important to purify and analyze different subcellular types separately in future investigations. A prominent site for heterologous LRRK2 phosphorylation is at serine 935 (pS935) that has been shown to be significantly reduced in PBMCs of affected G2019S carriers, distinguishing them from sPD, healthy G2019S carriers, and controls (Padmanabhan et al., 2020). Another study reported significantly elevated *in vitro* kinase activity of LRRK2 in PBMCs from *LRRK2* G2019S mutation carriers in comparison to non-carriers, while the pS935-LRRK2 level was increased in sPD compared to controls (Melachroinou et al., 2020). The molecular mechanisms that alter the regulation of S935 phosphorylation are not fully understood and need further clarification. Moreover, LRRK2 is sensitized to dephosphorylation by LRRK2 kinase inhibitors, with reduced pS935-LRRK2 that could be used as a marker of pharmacological LRRK2 inhibition (Dzamko et al., 2010). Whether the GTPase Rab10, the key substrate of LRRK2 that can be pharmacologically modified, could serve as a marker for target engagement is still inconclusive and needs further assessment (Steger et al., 2016; Thirstrup et al., 2017). At least, for discriminative purposes, Rab10 cannot be suggested since no difference could be found in blood cells between patients with PD and controls (Fan et al., 2018; Atashrazm et al., 2019). Although it has been challenging to directly measure LRRK2 in CSF, LRRK2 quantification in CSF could be obtained by an improved monoclonal antibody technique that has revealed significantly higher CSF LRRK2 levels in G2019S-PD compared to sPD, healthy controls, and non-manifesting G2019 carriers (Mabrouk et al., 2020). Auto-phosphorylation of LRRK2 at serine 1292 (pS1292) reflects kinase activity that is measurable in CSF, but possibly masked by saturation effects no changes in pS1292 levels could be detected in the CSF of G2019S carriers (Wang et al., 2017). As mentioned in the previous section, CSF protein analysis revealed the differential pha-synuclein profiles of individuals with LRRK2 mutation (Aasly et al., 2014; Vilas et al., 2016) that could result from the variable presence of LB pathology in PARK-LRRK2. No differences were found in terms of the AD-related proteins amyloid-β1-42, total-tau, or phospho-tau (Vilas et al., 2016). Fraser et al. (2013) demonstrated the presence of LRRK2 in urinary exosomes, small endosomal-derived vesicles released from cells to the periphery. Intriguingly, G2019S carriers with PD displayed higher pS1292 to the total LRRK2 ratio in urinary exosomes compared to non-carriers and even asymptomatic G2019S carriers, indicating the potential for risk prediction (Fraser et al., 2016). Further studies on LRRK2 exosomes will explore their biomarker potential in PD.

Regarding imaging biomarkers, positron emission tomography (*PET*) detected reduced tracer binding in presymptomatic LRRK2 mutation carriers compared to controls, with greater progression of dopaminergic dysfunction (Nandhagopal et al., 2008). In this PET study, multiple tracers were applied, including multitracer PET using 18F-6-fluoro-l-dopa, 11C-(±)-α-dihydrotetrabenazine and 11C-d-threo-methylphenidate. Dopamine transporter imaging with I-123 FP-CIT showed potential to monitor progression, although a certain degree of intraindividual variability limited its ability to predict phenoconversion (Sánchez-Rodríguez et al., 2021). Studies that investigate markers to discriminate LRRK2-PD from aPD are lacking.

## PRKN/PINK1

Given the common biological pathways affecting mitochondrial function and biomarker studies, including both *PRKN* and *PINK1* mutations, these genes are discussed together.

The majority of early-onset parkinsonism with autosomal-recessive inheritance is linked to mutations of the *PRKN/PARK2* gene (Kitada et al., 1998; Lücking et al., 2000). More than 130 disease-causing mutations have been identified throughout all 12 exons, including point mutations, exon rearrangements, and small deletions (Kasten et al., 2018). Whereas biallelic *PRKN* mutation results in overt disease, the question whether heterozygous mutations predispose to disease risk still remains an issue of debate (Huttenlocher et al., 2015; Lubbe et al., 2021; Yu et al., 2021). The encoded Parkin protein is an E3-ubiquitin ligase that transfers ubiquitin to protein substrates, thus targeting them to proteasomal degradation (Shimura et al., 2000; Moore, 2006). Moreover, Parkin regulates mitochondrial quality control and clearance of damaged mitochondria through mitophagy in concert with the other PD-linked gene *PINK1* (Narendra et al., 2012). In the Drosophila model, mutant *PRKN* caused a *degenerative phenotype with* flight muscle defects, *mitochondrial alterations, and abnormalities of dopaminergic neurons (Greene et al., 2003; Pickrell and Youle, 2015).*

PARK-PRKN is characterized by a slowly progressive disease course at an early age of the onset (mean, 31 years) (Khan et al., 2003; Kasten et al., 2018). Beyond the typical clinical triad, hyperreflexia can be present, and dystonia, often affecting the lower limb, can be an initial sign (Khan et al., 2003; Grünewald et al., 2013). The marked and sustained response to levodopa is complicated by frequently associated levodopa-induced motor fluctuations and dyskinesias (Clarimon et al., 2005). With regard to non-motor aspects, olfactory function and cognition are mostly well preserved (Khan et al., 2004; Alcalay et al., 2015; Abeliovich and Gitler, 2016). However, a multicenter, case-control study reported an earlier occurrence and a higher frequency and severity of impulsive-compulsive behaviors in PARK-PRKN (Morgante et al., 2016).

Mutations in the *phosphatase and tensin homolog (PTEN)-induced putative kinase 1* (*PINK1*) gene represent the second most frequent cause of autosomal-recessive inherited early-onset parkinsonism (Valente et al., 2004). The frequency of *PINK1* mutations ranges between 1 and 9%, with variations across different ethnic populations (Schulte and Gasser, 2011; Klein and Westenberger, 2012). Mutations are highly diverse and include missense, non-sense, frameshift mutations, and deletions. As with *PRKN*, the role of heterozygous *PINK1* mutation as a risk factor in PD has not yet been clarified (Krohn et al., 2020). PINK1 encodes a serine/threonine kinase involved in the regulation of the mitophagy pathway by activating ubiquitin and Parkin through phosphorylation (Valente et al., 2004; Narendra et al., 2010; Kane et al., 2014). Drosophila-lacking *PINK1* exhibited mitochondrial abnormalities and muscle and neuronal degeneration that could be rescued by *PRKN* overexpression but not *vice versa*, suggesting that *PINK1* acts upstream of Parkin in the shared pathway (Clark et al., 2006; Park et al., 2006; Yang et al., 2006).

In general, PARK-PINK1 shares similar features as PARK-PRKN regarding early AAO (mean, 32 years), slow disease progression, and excellent levodopa response, although associated with motor fluctuations and dyskinesias (Bonifati et al., 2005; Ibáñez et al., 2006; Kasten et al., 2018). Dystonia at the onset and hyperreflexia are also described in patients with *PINK1* mutation. However, non-motor symptoms are more pronounced. Hyposmia is commonly reported (Ferraris et al., 2009). Neuropsychiatric features are multifaceted, including mostly depression and anxiety but also traits of the schizophrenic spectrum and obsessive-compulsive personality disorder (Ephraty et al., 2007; Steinlechner et al., 2007; Ricciardi et al., 2014). Although cognitive function was thought to be less affected (Bonifati et al., 2005; Kasten et al., 2018), a recent study has observed a higher rate of cognitive dysfunction in *PINK1* mutations (Piredda et al., 2020).

Biomarkers for mitochondrial integrity are not only required to prove target engagement and drug-induced effects but also to identify patients who will most likely benefit from mitophagy-targeted treatments. PINK1 phosphorylates ubiquitin at a serine residue (pS65-Ub), which could reflect PINK1 activity. Autopsy studies have revealed elevated pS65-Ub levels in brain tissues of patients with sPD, DLB, and normal elderly controls, suggesting that pS65-Ub accumulates upon mitochondrial stress during disease or aging (Fiesel et al., 2015; Hou et al., 2018). In contrast, *PINK1/PRKN* mutants lacked pS65-Ub signals, consistent with their loss of function to label damaged mitochondria. More intriguingly, pS65-Ub signals detected by sensitive enzyme-linked immunosorbent assay were significantly reduced in blood plasma of patients with homozygous *PINK1*-mutation carriers compared to controls with heterozygous mutation and non-carriers (Watzlawik et al., 2020). Whether pS65-Ub levels can be used as a diagnostic or prognostic disease marker in blood needs to be confirmed in further investigations. Mitochondrial DNA (mtDNA) is closely related to mitochondrial function. Whereas reduced mtDNA copy numbers were reported in the substantia nigra of PD postmortem brains (Pyle et al., 2016), the findings in peripheral blood are inconclusive (Gui et al., 2015; Pyle et al., 2016; Davis et al., 2020). Circulating cell-free mtDNA (ccf-mtDNA) is a fragment of mtDNA released from cells in response to stress. Reduced ccf-mtDNA levels were reported in the CSF of patients with sPD (Pyle et al., 2015), showing an inverse correlation with treatment (Lowes et al., 2020). Notably, a recent study has observed higher ccf-mtDNA levels in the sera of *PRKN* and *PINK1* biallelic mutation carriers and affected heterozygotes compared to patients with sPD, possibly resulting from impaired mitophagy in PRKN/PINK1-associated PD (Borsche et al., 2020). As inflammation evidently contributes to PD pathogenesis and progression, peripheral inflammatory cytokines have been attributed biomarker potential. Among these, interleukin-6 (IL6) levels were elevated in blood samples from patients with PD (Qin et al., 2016), correlating with motor severity. A recent study has reported higher serum IL6 levels in patients with biallelic *PRKN/PINK1* mutations compared to healthy controls, whereas heterozygous *PRKN/PINK1* mutation carriers and sPD showed only a trend toward elevated IL6 levels, indicating a gene dosage effect (Borsche et al., 2020). Moreover, IL6 levels correlated only in affected *PRKN/PINK1* mutation carriers with disease duration. Mitochondrial dysfunction is also implied in the pathophysiology of a PD such as MSA and PSP (Nicoletti et al., 2021). Comparative studies exploring discriminative biomarkers of *PRKN/PINK1*-associated PD vs. aPD have not yet been conducted.

Imaging methods can be useful to monitor mitochondrial energy metabolism. Mitochondrial complex I activity can be assessed *in vivo* by the PET radiotracer ^18^F-BCPP-EF, which is reduced in a non-human primate model for PD (Tsukada et al., 2016) but could not be translated to patients with PD (Wilson et al., 2020). Alternatively, phosphorus magnetic resonance spectroscopy (^31^P-MRS) can visualize cerebral mitochondrial metabolism by measuring phosphorous-containing energy metabolites (Henchcliffe et al., 2008). Increased putaminal levels of high-energy phosphates distinguished homozygous *PINK1* mutation carriers from heterozygous mutation carriers and controls (Hilker et al., 2012). This finding was interpreted as compensatory mechanisms with respect to the impaired cellular stress resistance.

Two current trials testing vitamin K2 (Prasuhn et al., 2020) and coenzyme Q10 (Prasuhn et al., 2019) include *Parkin/PINK1* mutation carriers and use ^31^P-MRS for patient stratification and as a secondary end point.

## DJ1

DJ-1 is a widely expressed protein that participates in antioxidative stress mechanisms and mitochondrial regulation (Dolgacheva et al., 2019). Mutations in the corresponding gene *DJ-1* are responsible for an autosomal-recessive form of gPD (PARK-DJ-1). The phenotype of patients with PARK-DJ-1 consists of an early onset and slow progression of the disease and is, therefore, comparable with other autosomal-recessive forms like PARK-PRKN or PARK-PINK1. However, mutations in *DJ-1* are less frequent than PARK-PRKN or PARK-PINK1.

There are conflicting results regarding DJ-1 as a biomarker in the CSF and peripheral tissues, with increased and decreased DJ-1 levels in patients with sPD (Saito, 2014; Farotti et al., 2017). The same conflicting results were obtained when DJ-1 in the CSF was examined in the differential diagnosis of sPD vs. aPD (Farotti et al., 2017). A few studies with a low number of patients have investigated the concentration of DJ-1 in saliva in sPD with conflicting results. Therefore, DJ-1 in saliva does not play a role in daily routine (Farah et al., 2018). Another option is to determine the concentration of oxidized DJ-1 in blood or urine. Oxidized DJ-1 is induced by oxidative stress, which plays a major role in the pathophysiology of neurodegenerative diseases. Increased levels of oxidized DJ-1 have been described in patients with sPD compared to controls (Jang et al., 2018).

## Conclusion

A biomarker with a high sensitivity and specificity that could be introduced into clinical routine is currently not available. Biomarker research for PD has made great progress in recent years. Nevertheless, there is still a lack of robust biomarkers with high sensitivity and specificity that would satisfy diagnostic requirements. This is also true for monitoring disease progression and differential diagnosis of sPD, gPD, and aPD. Even for the most common monogenetic forms described here, this has not yet been achieved. This is true for the different forms of PD, including sPD, gPD, and aPD. Further research activity will be necessary to improve or find new techniques (e.g., RT-QuIC) and find the most suitable liquid biomaterial. The key to the diagnosis of PD, especially for future disease-modifying therapies, is likely to be a combination of different biomarkers with different modalities. In addition to peripheric biomarkers and CSF, imaging techniques (nuclear medicine, MRI, etc.) will play a crucial role.

## Author Contributions

LT and SK contributed to conception and design of the study. SK and EK wrote the first draft of the manuscript. All authors contributed to manuscript revision, read, and approved the submitted version.

## Conflict of Interest

The authors declare that the research was conducted in the absence of any commercial or financial relationships that could be construed as a potential conflict of interest.

## Publisher’s Note

All claims expressed in this article are solely those of the authors and do not necessarily represent those of their affiliated organizations, or those of the publisher, the editors and the reviewers. Any product that may be evaluated in this article, or claim that may be made by its manufacturer, is not guaranteed or endorsed by the publisher.
